# Persistence is key: unresolved immune dysfunction is lethal in both COVID-19 and non-COVID-19 sepsis

**DOI:** 10.3389/fimmu.2023.1254873

**Published:** 2023-09-26

**Authors:** Andy Y. An, Arjun Baghela, Peter Zhang, Reza Falsafi, Amy H. Lee, Uriel Trahtemberg, Andrew J. Baker, Claudia C. dos Santos, Robert E. W. Hancock

**Affiliations:** ^1^ Center for Microbial Diseases and Immunity Research, University of British Columbia, Vancouver, BC, Canada; ^2^ Department of Molecular Biology and Biochemistry, Simon Fraser University, Burnaby, BC, Canada; ^3^ Keenan Research Center for Biomedical Science and the Department of Critical Care, St. Michael’s Hospital, University of Toronto, Toronto, ON, Canada; ^4^ Department of Critical Care, Galilee Medical Center, Nahariya, Israel

**Keywords:** COVID-19, sepsis, immune dysfunction, gene expression, drug repurposing

## Abstract

**Introduction:**

Severe COVID-19 and non-COVID-19 pulmonary sepsis share pathophysiological, immunological, and clinical features, suggesting that severe COVID-19 is a form of viral sepsis. Our objective was to identify shared gene expression trajectories strongly associated with eventual mortality between severe COVID-19 patients and contemporaneous non-COVID-19 sepsis patients in the intensive care unit (ICU) for potential therapeutic implications.

**Methods:**

Whole blood was drawn from 20 COVID-19 patients and 22 non-COVID-19 adult sepsis patients at two timepoints: ICU admission and approximately a week later. RNA-Seq was performed on whole blood to identify differentially expressed genes and significantly enriched pathways. Using systems biology methods, drug candidates targeting key genes in the pathophysiology of COVID-19 and sepsis were identified.

**Results:**

When compared to survivors, non-survivors (irrespective of COVID-19 status) had 3.6-fold more “persistent” genes (genes that stayed up/downregulated at both timepoints) (4,289 vs. 1,186 genes); these included persistently downregulated genes in T-cell signaling and persistently upregulated genes in select innate immune and metabolic pathways, indicating unresolved immune dysfunction in non-survivors, while resolution of these processes occurred in survivors. These findings of persistence were further confirmed using two publicly available datasets of COVID-19 and sepsis patients. Systems biology methods identified multiple immunomodulatory drug candidates that could target this persistent immune dysfunction, which could be repurposed for possible therapeutic use in both COVID-19 and sepsis.

**Discussion:**

Transcriptional evidence of persistent immune dysfunction was associated with 28-day mortality in both COVID-19 and non-COVID-19 septic patients. These findings highlight the opportunity for mitigating common mechanisms of immune dysfunction with immunomodulatory therapies for both diseases.

## 1 Introduction

As of July 2023, the COVID-19 pandemic has infected >650 million and killed 6-18 million people globally (1, 2). COVID-19 patients who are admitted to the intensive care unit (ICU) have high mortality rates of up to 32%, with multiple organ failure causing the majority of these deaths (3). This is strikingly similar to severe sepsis, which is life-threatening organ failure caused by a dysregulated host response to infection (4), which is often a bacterial infection but can also be viral and fungal etiologies. Sepsis is estimated to kill 11 million people each year and be involved in 1 in 5 deaths globally (5), having average 30-day mortality rates of 24.4% for sepsis and 34.7% for septic shock in North America, Europe, and Australia (6), and even higher in lower and middle income countries (7). Due to similarities in immune dysfunction, endothelial disruption, cytokine levels, gene expression, and long-term consequences, there is a growing consensus that severe COVID-19 should be classified and treated as a form of viral-associated sepsis (8). We recently have shown that our sepsis endotypes can accurately classify COVID-19 patients based on severity (9, 10) and that contemporaneous severe COVID-19 and non-COVID-19 sepsis patients converge into transcriptionally indistinguishable mechanisms after a week in the ICU (11).

Despite high mortality rates, no specific treatment for sepsis is available other than antibiotics and supportive management (*e.g.*, fluid resuscitation) (12). The lack of treatments is not due to a lack of effort, as exemplified by over 30 years of failed sepsis clinical trials (13). However, these clinical trials focused only on the inflammatory aspect of sepsis, while it has become evident that a concurrent immunosuppressive arm of sepsis is also occurring, potentially as a means to limit life-threatening inflammation (14). Thus, simply using immunosuppressive/anti-inflammatory therapies for sepsis patients can exacerbate this immune dysregulation and exposes the patient to lethal opportunistic pathogens. These concurrent immunosuppression and inflammation processes are part of a syndrome termed “Persistent Inflammation, Immunosuppression, and Catabolism Syndrome” (PICS), that is proposed to occur in septic ICU patients with disastrous consequences including recurrent nosocomial infections, poor wound healing, inability of self-care, and eventual death (15), but has not been defined mechanistically. Further research into the specific mechanisms underlying why patients progress to death is critically needed for development of targeted therapies for sepsis. Previous work in the Hancock Lab found five endotypes at early disease presentation, two of which were correlated with higher mortality rates (Neutrophilic-Suppressive and Inflammatory), and a cross-cutting mortality signature was identified (9). Collectively these suggest both mechanistically-variable and conserved pathways are involved in mortality. In addition to these genetic biomarkers, many blood biomarkers have been shown to predict disease severity and mortality in COVID-19, such as C-reactive protein, procalcitonin, D-dimer, interleukin-6, lactate dehydrogenase, ferritin, plasma Gas6, lymphopenia, and thrombocytopenia (16–19), with many previously investigated in sepsis (20).

There has been an unprecedented level of scientific interest and funding, as well as success, for the treatment of COVID-19, resulting in clinical trials discovering effective therapies. The use of immunomodulators such as corticosteroids (21), tocilizumab (22), and baricitinib (23) has shown promising effects on reducing mortality and other severity metrics. If mortality mechanisms are shared between COVID-19 and sepsis patients, these promising results will likely have applications to sepsis therapeutics. Nevertheless, both sepsis and COVID-19 are highly dynamic diseases (11, 14, 24), requiring analysis of multiple timepoints to fully understand disease trajectories and uncover additional pathophysiology that cannot be detected from a single timepoint.

In this study, we aimed to identify shared mechanistic trajectories related to mortality in severe COVID-19 patients and contemporaneous non-COVID-19 sepsis patients. Shared gene expression changes over time could underscore common mechanisms of injury and/or repair, with broad therapeutic implications. We showed that persistent immune dysfunction was highly associated with patients who died regardless of SARS-CoV-2 positivity, a finding that was replicated in other public datasets of COVID-19 and sepsis patients. Based on this finding of persistence, we also identified potential treatments targeting these persistent genes in both COVID-19 and non-COVID-19 sepsis.

## 2 Materials and methods

### 2.1 Study design and sample collection

Between March 2020 and February 2021, the prospective observational “COVID-19 Longitudinal Biomarkers of Lung Injury” (COLOBILI) study consented and enrolled 42 ICU adult (>18 years) patients with respiratory deterioration from suspected COVID-19 at St. Michael’s Hospital (Toronto, Canada) (**Table 1**). Whole blood (2.5 mL) was drawn into PaxGene Blood RNA tubes (BD Biosciences) at admission (Day 1, D1) and Day 7 (D7) in the ICU. After enrollment, 20 patients were identified to be SARS-CoV-2 PCR positive (but blood culture negative at both timepoints), and the remaining 22 SARS-CoV-2 PCR negative patients had ≥2 negative PCR tests. All patients satisfied Sepsis-3 criteria for sepsis (suspected/confirmed infection with a SOFA score ≥2 at ICU admission) (4). After the second blood draw, nine patients (4 SARS-CoV-2 positive, 5 negative) died within 28 days in the ICU. Samples were frozen and transported to Vancouver, Canada, for RNA extraction (PAXgene Blood RNA Kit; Qiagen) followed by RNA-Seq. Whole blood from 5 healthy controls from Vancouver, Canada were processed alongside the patient samples. Further details on study design and RNA-Seq methodology can be found in our previously published protocol (11).

**Table 1:** Patient demographics of ICU patients, separated by mortality.

Clinical Variables	Non-Survivors (9)	Survivors (33)	P-value
Patient Demographics			
Age	62.4 ± 16.4 (9)	59.2 ± 15 (33)	0.540
Sex (Male)	88.9% (8/9)	72.7% (24/33)	0.416
SARS-CoV-2 (Positive)	44.4% (4/9)	48.5% (16/33)	1.000
Duration of ICU stay (Days)	14.3 ± 4.4 (9)	27.8 ± 23.3 (33)	0.149
Steroids During Hospitalization (Yes)	55.6% (5/9)	51.5% (17/33)	1.000
Body Mass Index	23.7 ± 4.8 (9)	31.1 ± 10.2 (33)	**0.021**
Illness Pre-Admission (Days)	12.5 ± 12.2 (6)	6.5 ± 7.5 (29)	0.342
Antibiotics Used Pre-Admission (Yes)	0.0% (0/9)	9.1% (3/33)	1.000
Smoker (Yes)	33.3% (3/9)	21.2% (7/33)	0.660
Race			0.771
African origins	11.1% (1/9)	9.1% (3/33)	
Asian origins	22.2% (2/9)	30.3% (10/33)	
European origins	0% (0/9)	6.1% (2/33)	
Latin, Central, South American origins	0% (0/9)	3% (1/33)	
North American Aboriginal origins	0% (0/9)	12.1% (4/33)	
Other North American origins	33.3% (3/9)	21.2% (7/33)	
Unknown	33.3% (3/9)	18.2% (6/33)	
Respiratory Comorbidities			
Asthma (Yes)	11.1% (1/9)	9.1% (3/33)	1.000
Obstructive Sleep Apnea (Yes)	0.0% (0/9)	18.2% (6/33)	0.312
Pneumonia (Yes)	0.0% (0/9)	12.1% (4/33)	0.561
COPD (Yes)	0.0% (0/9)	15.2% (5/33)	0.567
Bronchiectasis (Yes)	0.0% (0/9)	3.0% (1/33)	1.000
Previous Pulmonary Surgery (Yes)	0.0% (0/9)	6.1% (2/33)	1.000
Day 1 ICU Variables			
SOFA Score	12.2 ± 2.3 (9)	8.8 ± 3.1 (33)	**0.006**
Glasgow Coma Score	3.9 ± 0.3 (9)	2.5 ± 1.6 (33)	**0.017**
Respiratory SOFA Score component	2.6 ± 0.7 (9)	2.6 ± 0.9 (31)	0.578
Admission APACHE II Severity Score	30.3 ± 6.6 (9)	24.2 ± 7.7 (33)	0.063
PaO_2_/FiO_2_ Ratio	194 ± 85 (9)	187 ± 86 (31)	0.582
On Mechanical Ventilation (Yes)	100.0% (9/9)	81.8% (27/33)	0.312
Given Antibiotics (Yes)	88.9% (8/9)	87.9% (29/33)	1.000
Blood Culture Positive (Yes)	11.1% (1/9)	12.1% (4/33)	1.000
White Blood Cells (10^3^ cells/µL)	15.7 ± 7.2 (9)	9.9 ± 5.4 (33)	**0.018**
Neutrophils (10^3^ cells/µL)	13.8 ± 6.8 (9)	8.3 ± 5.2 (32)	**0.026**
Lymphocytes (10^3^ cells/µL)	0.9 ± 0.5 (9)	0.9 ± 0.7 (32)	0.765
Monocytes (10^3^ cells/µL)	0.5 ± 0.4 (9)	0.5 ± 0.4 (32)	0.329
Eosinophils (10^3^ cells/µL)	0 ± 0 (9)	0.1 ± 0.2 (32)	**0.021**
Platelets (10^3^ platelets/µL)	215.7 ± 121.9 (9)	197.9 ± 102 (33)	0.830
Fibrinogen (g/L)	4.5 ± 1.4 (3)	4.1 ± 2.2 (9)	0.579
D-Dimer (ng/mL)	2373 ± 2296 (3)	2688 ± 1761 (6)	0.604
C-Reactive Protein (mg/L)	44.6 ± 39.6 (2)	122 ± 76.3 (10)	0.107
Lactate (mmol/L)	3 ± 1.9 (9)	1.6 ± 0.8 (28)	0.055
Day 7 ICU Variables			
SOFA Score	9 ± 5.2 (9)	6.1 ± 3.7 (33)	0.161
Glasgow Coma Score	3.7 ± 0.5 (9)	2.1 ± 1.3 (33)	**0.001**
Respiratory SOFA Score component	2.6 ± 0.5 (7)	2.7 ± 0.7 (23)	0.637
PaO_2_/FiO_2_ Ratio	218.3 ± 62.6 (7)	177.7 ± 69 (23)	0.230
On Mechanical Ventilation (Yes)	100.0% (9/9)	72.7% (24/33)	0.166
Given Antibiotics (Yes)	77.8% (7/9)	57.6% (19/33)	0.442
Blood Culture Positive (Yes)	0.0% (0/9)	0.0% (0/33)	1.000
White Blood Cells (10^3^ cells/µL)	13.1 ± 2.8 (9)	9.9 ± 3.7 (32)	**0.019**
Neutrophils (10^3^ cells/µL)	11.1 ± 2.7 (9)	7.5 ± 3.3 (32)	**0.007**
Lymphocytes (10^3^ cells/µL)	0.9 ± 0.4 (9)	1.3 ± 0.7 (32)	0.244
Monocytes (10^3^ cells/µL)	0.7 ± 0.5 (9)	0.7 ± 0.3 (32)	0.987
Eosinophils (10^3^ cells/µL)	0 ± 0 (9)	0.2 ± 0.2 (32)	**0.002**
Platelets (10^3^ cells/µL)	178.8 ± 127.7 (9)	312.2 ± 183 (31)	**0.033**
Fibrinogen (g/L)	1.5 ± 0.8 (3)	6.5 (1)	0.371
D-Dimer (ng/mL)	2061.1 ± 2907.5 (2)	NA	NA
C-Reactive Protein (mg/L)	515.9 ± 411.7 (2)	422 (1)	1.000
Lactate (mmol/L)	9.8 ± 15.9 (6)	1.5 ± 0.5 (12)	0.372

For categorical variables, significance was tested using the Chi-squared test with Yates’s correction, or Fisher’s exact test if any expected value was <5, and the percentage and fraction of patients fitting the category is displayed. For continuous variables, the Wilcoxon Rank-Sum test was used, and the mean ± standard deviation of the variable is displayed, with the number of patients assessed in brackets. Remdesivir was used in one patient and tocilizumab was used in two patients, all of whom were survivors. Bolded p-values indicated significant differences (p <0.05).

### 2.2 Bioinformatic and statistical analysis

Differentially expressed (DE) genes were identified between different patient subgroups using the *DESeq2* package (25), with DE genes defined as having an adjusted p-value <0.05 (Benjamini-Hochberg correction) and an absolute fold change ≥1.5. The DESeq2 model included sex and batch as confounders to adjust for. Up-/down-regulated DE genes were used to identify significantly enriched pathways/gene sets from the Reactome pathways database (26) using gene-pair overrepresentation analysis with *SIGORA* (Bonferroni adjusted P-value <0.001), and from the Molecular Signatures Database Hallmark gene sets (27) using overrepresentation analysis from *clusterProfiler (* 28) (Benjamini-Hochberg adjusted P-value <0.05). Significantly enriched pathways/gene sets indicated key dysregulated biological processes. Gene-drug interactions from the Drug Signatures Database (29) were also analyzed in a similar fashion as pathway enrichment using *clusterProfiler*. Pathway plots, volcano plots, and fold change heatmaps were generated using *pathlinkR* (https://github.com/hancockinformatics/pathlinkR). Confirmatory studies were done using publicly available datasets GSE196117 (30) and GSE161918 (28).

## 3 Results

### 3.1 Non-survivors had substantially more persistent genes compared to survivors

To understand the determinants of gene expression variation, unsupervised principal component analysis (PCA) was used. PCA summarizes gene expression variation into individual principal components (PCs), where the PCs are numbered based on the fraction of variation they describe. PCA of the ICU samples (**Figure S1A**) demonstrated that eventual mortality, disease severity (SOFA score), and sample collection time were significantly correlated with PC1 (the PC with the largest percentage of variation, 22.5%), while COVID-19 status was only significantly correlated with the smaller PC3 (9.1% of variation) (**Figure S1B**). This suggested that the pathogen had a smaller impact on overall gene expression variation when compared to eventual mortality in the ICU and disease stage. The importance of mortality and sampling time to gene expression variation prompted a thorough investigation of gene expression trajectories in survivors and non-survivors.

**Figure 1:**
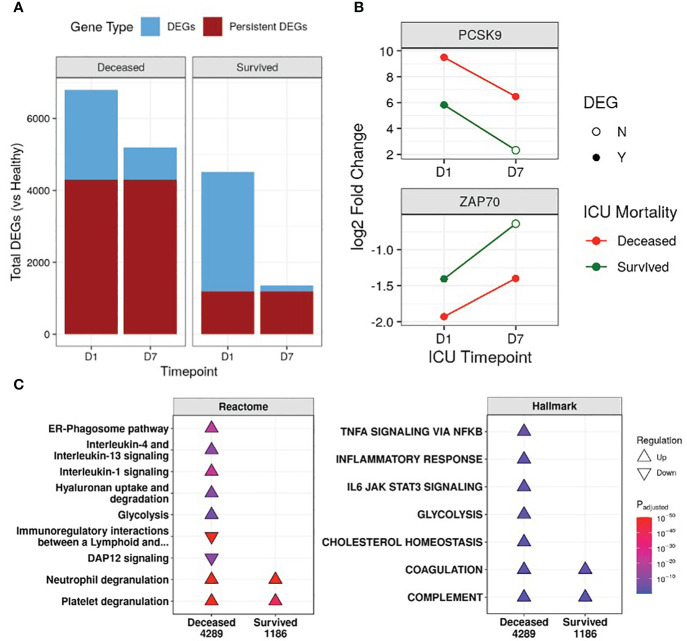
Non-survivors had substantially more persistent genes than survivors. **(A)** Number of differentially expressed genes (DEGs) at either Day 1 (D1) or Day 7 (D7) in the ICU for non-survivors and survivors, compared to healthy controls. The fraction of persistent DEGs (DEGs that were up/down-regulated at both timepoints) among all DEGs is highlighted. **(B)** Examples of persistently upregulated (*PCSK9*) or persistently downregulated (*ZAP70*) genes only found in non-survivors are shown. Empty circles indicate that the gene was no longer a DEG at that timepoint. **(C)** Eventually deceased patients had persistent dysfunction of immune and cellular pathways. A subset of enriched Reactome pathways and Hallmark gene sets from persistently upregulated (Δ) and downregulated (∇) genes in patients who eventually were deceased or survived are shown. The total numbers of persistent genes in each comparison are under each label. All enriched pathways and gene sets can be found in **Figures S5** and **S6**. Pathway plots were generated using *pathlinkR* (https://github.com/hancockinformatics/pathlinkR).

First, the transcriptional profiles of survivors and non-survivors were compared to healthy controls to determine whether there were “persistent” genes, *i.e.*, DE genes that remained consistently up/downregulated throughout the first week of ICU compared to healthy controls, indicating unresolved dysfunction (**Figure 1B**). Non-survivors had 3.6-fold the number of persistent genes when compared to survivors (4,289 vs 1,186) (**Figure 1A**), and this trend of more persistent genes in non-survivors was present even when separating into COVID-19 and non-COVID-19 sepsis patients (**Figure S2C**). Notable persistent genes found only in non-survivors included immune genes such as *IL1R1/2*, *IL4R*, *IRAK3*, *ZAP70*, and the sepsis mortality gene *PCSK9* (29).

Since persistence suggested potential long-term defects that might be related to genetic differences, we compared these persistent genes to four genome-wide association studies of sepsis (31, 32) and COVID-19 severity (33, 34) (**Table S2**). Fifty persistent genes overlapped with GWAS identified genes, with almost all (46/50) found in non-survivors (**Table S2**). These included sepsis severity genes such as *PCSK9*, *CACNA2D2*, and *HEMK1 (* 31, 32), and COVID-19 severity genes such as *IL10RB, TYK2*, and *F8 (* 33, 34) (**Table S2**). Interestingly, *ICAM1* (intercellular adhesion molecule 1) was found in GWAS studies for both sepsis and COVID-19 and was also a persistently upregulated gene only in non-survivors, suggesting persistent dysregulation of this gene may be involved in worse outcomes for both diseases. Increased surface expression of ICAM-1 on leukocytes occurs during inflammation to promote leukocyte adhesion and extravasation (35). Circulating ICAM-1, a marker of endothelial damage, has been documented to be elevated in sepsis and COVID-19 patients, with higher levels associated with increased severity (36–38).

Persistence also implicated epigenetic regulation; we found 34 genes that overlapped between persistent genes in non-survivors and differentially methylated genes of sepsis patients identified in a previous study (39) and those of COVID-19 patients (40, 41) (**Figure S3A**), suggesting the potential presence of epigenetic switches that might be responsible for persistent dysregulation of genes. Notably, these overlapping genes were mostly immune-related genes, such as *CD177*, *CD3D*, and *S100P* (**Figure S3B, C**). Lastly, the concept of substantially more persistent genes in patients with worse outcomes was also replicated/validated in 91 samples from two external longitudinal datasets of critically ill sepsis (30) and COVID-19 patients (42) (**Figure S4**).

### 3.2 Unresolved immune dysfunction was associated with eventual ICU mortality

We next investigated the functional consequences of persistent genes in non-survivors. Pathway enrichment identified 46 and 10 unique pathways enriched by persistent genes in non-survivors and survivors, respectively, with 6 shared pathways (**Figure S5**). In non-survivors, these unique Reactome pathways included pathways from the “Immune System”, “Metabolism”, “Metabolism of RNA”, “Metabolism of proteins”, and “Organelle biosynthesis and management” categories, suggesting persistent dysfunction in multiple aspects of cellular function (**Figure S5**). Specifically, in non-survivors, interleukin (IL) and inflammatory pathways (“IL-1 signaling”, “IL-4/13 signaling”, “ER-phagosome pathway”) were persistently upregulated, while adaptive immune activation pathways such as “Immunoregulatory interactions between a lymphoid and a non-lymphoid cell” and “DAP12 signaling” were persistently downregulated (**Figure 1C**). This indicated enduring immune dysfunction in non-survivors, but not in survivors, and was recapitulated by the Hallmark gene sets “Inflammatory Response”, “TNFα signaling via NF-kB”, and “IL-6 JAK STAT3 signaling” in non-survivors (**Figure 1C**). Interestingly, the gene sets “Cholesterol homeostasis” and “Glycolysis” and the pathway “Hyaluronan uptake and degradation” were also persistently upregulated only in non-survivors, highlighting known metabolic dysfunctions associated with worse outcomes for sepsis (43–45). The “Coagulation” and “Complement” gene sets and the “Neutrophil degranulation” and “Platelet degranulation” pathways were persistently upregulated in all patients (**Figure 1C**), suggesting shared immune and coagulation dysfunction among all patients in the ICU.

Many of these immune pathways (“IL-1 signaling”, “IL-4/13 signaling”, “Neutrophil degranulation”, and “Immunoregulatory interactions”) were also enriched by persistent genes in the two validation datasets of COVID-19 and sepsis patients (**Figure S4**), as well as in persistent genes of non-survivors even after splitting into COVID-19 and non-COVID-19 sepsis patients (**Figure S2D**), suggesting shared mechanisms of mortality between these two diseases. This idea of shared mortality mechanisms was further supported by the comparison of COVID-19 to non-COVID-19 sepsis non-survivors at D1 and D7. There were 275 DE genes at D1 between COVID-19 and non-COVID-19 sepsis non-survivors (mostly enriching for antiviral pathways, reflective of the pathogen-specific response), which dropped to just one DE gene at D7, suggesting a convergence to shared mortality mechanisms over time (**Figures S2A, B**).

**Figure 2:**
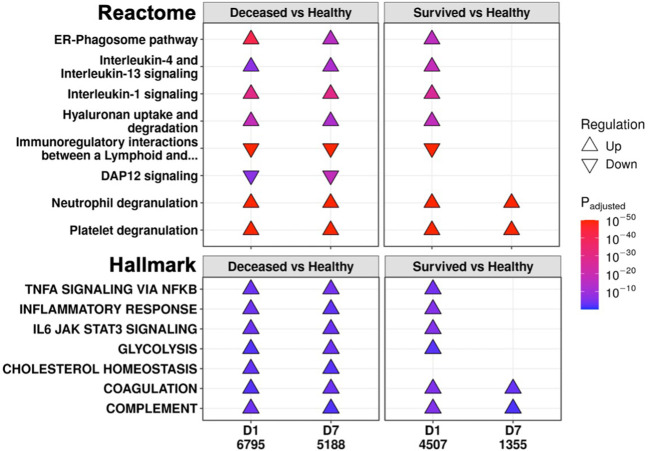
Eventually deceased patients had unresolving immune dysfunction. A subset of significantly enriched Reactome pathways (top) and Hallmark gene sets (bottom) using differentially expressed (DE) genes from comparing eventually deceased or surviving patients to healthy controls at Day 1 (D1) and Day 7 (D7). The total numbers of DE genes in each comparison are under each label. All enriched pathways and gene sets shown in **Figures S6** and **S7**. Pathway plots were generated using *pathlinkR*.

**Figure 3:**
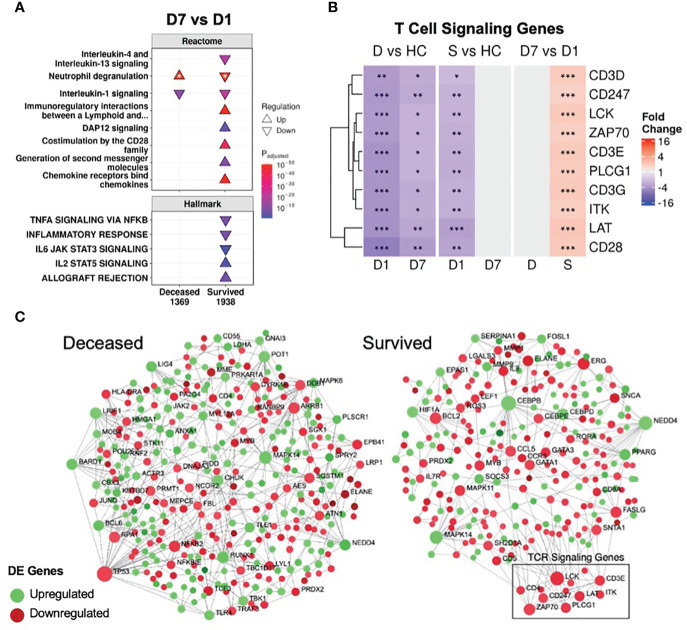
Resolution of inflammation and adaptive suppression occurred only in survivors. **(A)** A subset of significantly enriched Reactome pathways and Hallmark gene sets using differentially expressed (DE) genes over time. The total numbers of DE genes in each comparison are under each label. All enriched pathways and gene sets shown in **Figures S6** and **S7**. For one pathway, both directions were enriched (indicated by *); the direction with the lower adjusted p-value (more significantly enriched) is shown. **(B)** Persistent suppression of T cell signaling genes was observed in non-survivors. D = deceased, S = survived, HC = healthy controls. Shading in the heatmap represents fold change. Only DE genes are shown. Significance values are derived from DESeq2 results: *** = p<0.001, ** = p<0.01, and * = p<0.05. Pathway plots and heatmaps were generated using *pathlinkR*. **(C)** Network analysis of DE genes over time in survivors and non-survivors. Zero-order (*i.e.*, only dysregulated nodes) functional protein-protein interactions (PPI) networks were drawn using NetworkAnalyst. Dots represent nodes (genes and their protein products) and are colored for directionality. Lines joining the dots represent known PPI from InnateDB. Differential expression of TCR signaling genes was not seen in non-survivors but these genes were upregulated over time in survivors (boxed area).

To further investigate persistent immune dysfunction, enrichment using all DE genes of survivors and non-survivors vs. healthy controls, not just persistent genes, was performed (**Figure 2**). This analysis demonstrated that while many of these pathways were enriched in both survivors and non-survivors at D1, they were only enriched in non-survivors at D7, suggesting that initially, all patients had immune dysfunction, but only survivors appeared to resolve their immune dysfunction and accompanying metabolic dysfunction (*e.g.*, glycolysis, hyaluronan metabolism, cholesterol metabolism) by D7 (**Figure 2**). Inflammatory resolution in survivors was also supported by enrichment results from DE genes that changed over time (**Figure 3A**). For example, the “Inflammatory response” gene set was upregulated in all patients at D1 compared to healthy controls, but only in non-survivors at D7 (**Figure 2**). This gene set was downregulated over time only in survivors (**Figure 3A**). Overall, this enrichment pattern highlighted that the general inflammatory response was resolved by D7 in survivors. A similar pattern underscoring persistent upregulation of the “IL-1 signaling” and “IL-4/13 signaling” pathways and the inflammatory gene sets “TNFα signaling via NF-kB” and “IL6 JAK STAT3 signaling,” was also observed only in non-survivors (**Figures 2**, **3A**), again supporting an unresolved inflammatory state in non-survivors but resolution in survivors.

In conjunction with resolving inflammation, survivors also resolved adaptive immune suppression, which was likely key to their survival, since a weakened adaptive immune response is strongly implicated in sepsis and COVID-19 severity/mortality (46, 47). The “Immunoregulatory interactions” pathway (which contains B-cell and T-cell activation genes) was persistently downregulated in non-survivors (**Figures 1C**, **2**); however, this pathway was downregulated in survivors only at D1. Time analysis showed that only in survivors was there upregulation over time of this pathway, as well as T-cell signaling pathways (“Co-stimulation by the CD28 family”, “Generation of second messenger molecules”) and adaptive immunity gene sets (“IL2 STAT5 signaling”, “Allograft rejection”) (**Figure 3A**), consistent with resolution of adaptive immune suppression in survivors. A closer examination of canonical T-cell signaling genes supported this observation: genes such as *CD3D/E/G*, *CD247*, *ZAP70*, *LCK*, *LAT* and *ITK* were all persistently downregulated in non-survivors, but were initially downregulated and increased back to normal levels in survivors by D7 (**Figure 3B**). *CD3D* was also a shared differentially methylated gene observed in sepsis (39) and COVID-19 (40, 41), suggesting potential epigenetic regulation related to T-cell dysfunction (**Figure S3C**). Lastly, using NetworkAnalyst (48), a functional protein-protein interaction network was created using DE genes over time, revealing that only survivors had a cluster of upregulated T-cell signaling genes (**Figure 3C**). Combined, these findings highlighted sustained adaptive immune dysfunction as a key aspect in patients who eventually died, and restoration of such adaptive deficits was only observed in survivors.

Various confounders that could affect these adaptive immunity findings were investigated. Corticosteroid use, which can affect leukocyte function, was unlikely to affect this result, since survivors and non-survivors did not have significant differences in the rate of corticosteroid use (**Table 1**). Differences in leukocyte populations were observed between survivors and non-survivors (**Table 1**), so differential expression analysis was performed after correcting for cellular composition estimated by *CIBERSORTx*, a cell deconvolution method based on gene expression data (49). Fold changes for genes before and after correction were highly significantly correlated (**Figure S8A**), suggesting that gene expression variation was not substantially driven by differences in cell proportions. Indeed, key T cell signaling genes were still persistently suppressed in non-survivors and resolved over time only in survivors after correction (**Figure S8B**).

When individually analyzing COVID-19 and non-COVID-19 sepsis patients, various adaptive immune pathways, including “Generation of second messenger molecules”, “Costimulation by the CD28 family”, and “Immunoregulatory interactions”, were still upregulated over time in survivors, but not non-survivors, of both groups, while “IL-1 signaling” was downregulated over time in survivors of both groups (**Figure S9**). This emphasized a common adaptive immune recovery and inflammation resolution mechanism associated with ICU survival. Interestingly, only COVID-19 survivors had downregulation of antiviral pathways over time (**Figure S9**), suggesting that resolution of elevated antiviral responses was a unique part of survival trajectories in viral sepsis from COVID-19.

While the numbers of survivors and non-survivors were somewhat unbalanced (reflecting expected mortality rates of sepsis in the ICU), performing a subset analysis matching equal numbers of survivors and non-survivors (by age, sex, COVID-19 status, and sequencing batch) resulted in similar results (**Figure S10**), with substantially more persistent genes in non-survivors (4,366 vs. 831). Again, persistent upregulation of “IL-1 signaling” and “IL-4/13 signaling” and persistent downregulation of “Immunoregulatory interactions” was seen in non-survivors only, suggesting that imbalance was not substantially affecting the results.

### 3.3 Non-survivors had persistent enrichment of the mortality signature and were “locked-in” to more severe sepsis endotypes

**Figure 4:**
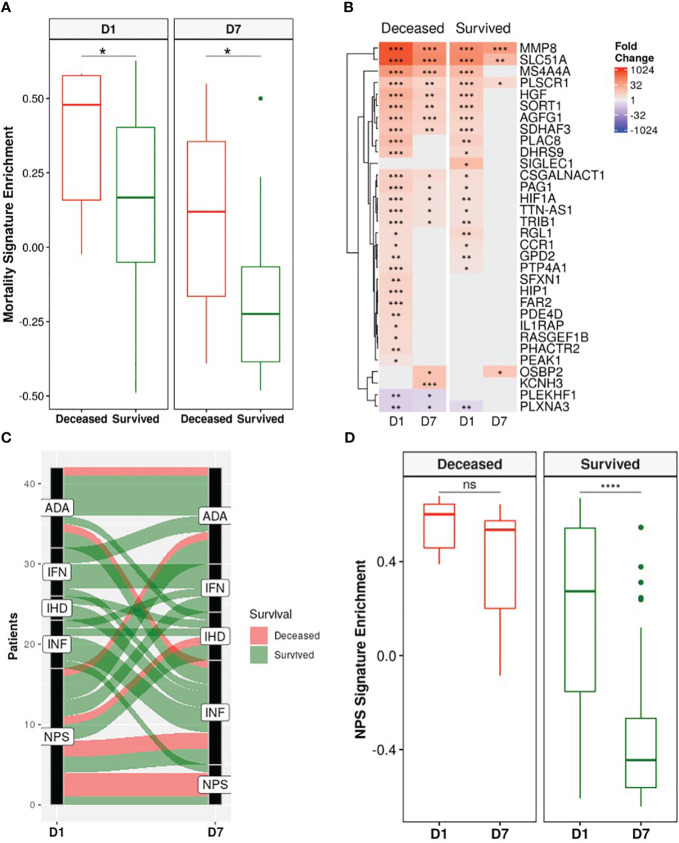
Non-survivors had persistent enrichment of the mortality signature and were “locked-in” to more severe sepsis endotypes. **(A)** The gene set variation analysis (GSVA) enrichment score of our published 38-gene mortality signature (9) was significantly higher in eventually deceased patients compared to survivors at both D1 and D7 (Wilcoxon rank-sum test, * = p<0.05). **(B)** Fold changes of mortality signature genes in eventually deceased patients and survivors relative to healthy controls. Shading in the heatmap represents fold change. Only DE genes are shown. Non-survivors had 15 persistently dysregulated (differentially expressed at D1 and D7) mortality signature genes compared to only 3 in survivors. **(C)** Eventually deceased patients were “locked in” to severe endotypes. Five endotypes were derived from differentially expressed genes in a cohort of emergency room sepsis patients: Neutrophilic-Suppressive (NPS), Inflammatory (INF), Interferon (IFN), Adaptive (ADA), and Innate Host Defense (IHD) (9). Each patient was classified into an endotype based on which of the five endotypes had the highest GSVA enrichment score. An alluvial graph demonstrating transition of each patient’s endotype between D1 and D7 is shown. **(D)** NPS GSVA enrichment scores were persistently high in eventually deceased patients but significantly decreased in survivors (pair-wise Wilcoxon rank-sum test, * = p<0.05; ** = p<0.01, *** = p<0.001, **** = p<0.0001, ns = not significant).

A recently published a 38-gene mortality signature derived from early (emergency room) sepsis patients (9); was then assessed in these ICU patients. Gene set variation analysis (GSVA), an unsupervised gene set enrichment calculation methodology (50), showed that enrichment scores of this signature were significantly and persistently higher in non-survivors at both D1 and D7 compared to survivors (**Figure 4A**). Moreover, there was greater overlap of the mortality signature with persistent genes of non-survivors (15/38) than those of survivors (3/38) (**Figure 4B**). This again indicated that persistent cellular dysfunction in non-survivors was associated with mortality in both sepsis and COVID-19.

We next investigated the utility of five endotypes previously identified in emergency room sepsis patients (9) and validated in COVID-19 patients (10). Two of the five endotypes were associated with worse outcomes: Neutrophilic-Suppressive (NPS) and Inflammatory (INF). Consistent with this, the majority (7/9) of eventually deceased patients started as NPS, and most continued as NPS or transitioned into the other severe outcome endotype INF, with 6/9 deceased patients fitting into either the NPS or INF endotypes at D7 (**Figure 4C**). Furthermore, NPS signature enrichment scores remained persistently elevated over time in non-survivors, but in survivors significantly decreased over time (**Figure 4D**), suggesting that patients who eventually died might be “locked-in” to the more severe endotypes, and that these emergency room sepsis endotypes are still useful for stratifying patients at later timepoints in the ICU. Ultimately, the utility of these sepsis mortality signatures and endotypes in this group of ICU patients further confirmed shared mechanisms of mortality between sepsis and COVID-19.

### 3.4 Repurposed drugs were identified to target persistent genes and hub genes

We have shown that persistent gene expression changes, reflective of immune dysfunction, were highly associated with eventual mortality; however, survivors were able to resolve this dysfunction. Therefore, currently approved medications that can target the persistent genes in non-survivors to resolve this dysfunction might lead to successful and rapid repurposing of drugs for sepsis and COVID-19 therapy. To identify drugs that resolve persistent dysfunction, two systems-biology approaches were utilized, rather than focusing on targeting single genes.

The first approach was to use the Drug Signatures Database (DSigDB) (29), which is a repository of FDA-approved medications and their interactions (*e.g.*, antagonism) with genes/proteins. Using this approach considers the overall systems-level cellular effect of a drug rather than its effect on just one target. These drug-gene sets were enriched using upregulated persistent genes only in non-survivors to identify possible inhibitors for these processes (**Table 2**). There was significant enrichment of numerous anti-inflammatory drugs including aspirin, sulfasalazine, and numerous corticosteroids including dexamethasone, likely targeting persistently upregulated inflammatory pathways. Surprisingly, other enriched drugs included antipsychotics (thioridazine and pimozide), the anti-arrhythmic agent flecainide, and various anti-infectives (mefloquine, ribavirin).

**Table 2:** Enriched drug signatures from the Drug Signatures Database (DSigDB).

Drug	Class	P_adj_	Genes	Persistent Genes in Drug-Gene set
Aspirin	Non-steroidal anti-inflammatory drug	2.13E-05	86	*ACTB, ALDOA, ATP6V0B, B4GALT1, BAX, CAPNS1, CAPZA1, CAPZA2, CCND3, CD44, CDC42, CEBPB, CFLAR, CHMP1A, CSF3R, CSTA, DENND5A, ETS2, FCER1G, FGR, FLII, FLOT2, FOS, FPR1, FPR2, G6PD, GABARAP, GHITM, GNB2, HCK, HCLS1, HGF, HSPA1A, ICAM1, IL18, IL1R2, JUN, LDLR, LYN, MAP2K2, MAP4K4, MAPK1, MAPK3, MLF2, NFKB1, NFKBIA, NFKBIZ, NPEPPS, OSM, PAG1, PCBP1, PDLIM7, PEF1, PFKFB3, PFN1, POR, PPIB, PPP4C, PRKCB, PXN, RAC1, RALB, RHOG, S100A11, SAT1, SELL, SERPINA1, SERPINB1, SERPINB2, STAT3, STAT5B, TACC3, TBC1D10B, TBXAS1, TCIRG1, TGFB1, THBS1, TIMM17B, TMSB4X, TNFRSF1A, TPD52L2, TXNRD1, TYK2, UBTD1, VAV1, WBP2*
Budesonide	Corticosteroid	1.85E-03	20	*ACSL1, AREG, CEBPD, CPD, CYP1B1, DAPK3, DUSP1, FKBP5, IFNGR1, IL1R2, IL4R, IRAK3, MAP3K3, NFE2L2, NFKBIA, PER1, SAMSN1, SAP30, STAT3, TLR2*
Thioridazine	Antipsychotic	1.85E-03	61	*ARFGAP3, ARHGEF2, ARID3B, ASAH1, ATXN1, BCL3, CARS1, CD44, CD55, CEBPB, CPNE3, CSGALNACT1, CSGALNACT2, DENND3, DNAJC3, DUSP1, EPAS1, ETS2, FAM114A1, FOS, FOSL2, FPR1, GADD45B, GDPD3, GK, H3C6, HRH2, ICAM1, IDI1, IER2, IER3, IL1RL1, IRS2, JUN, JUNB, KDM7A, KLF5, KLF6, LDLR, MAP2K6, MNT, MSMO1, NDRG1, NEAT1, NFIL3, NFKBIA, NIBAN1, PIM1, PLEC, PNRC1, PRL, S100P, SAT1, SLC38A2, STX3, SVIL, TNIP1, ULK1, WBP2, WIPI1, ZFP36*
Halcinonide	Corticosteroid	1.85E-03	13	*ACSL1, AREG, CD163, CPD, DUSP1, FKBP5, FPR1, IL1R2, NFE2L2, PER1, SAMSN1, THBS1, TPST1*
Podophyllotoxin	Antiviral	1.85E-03	77	*APOBR, AREG, ARID3B, ARRB2, ASAH1, ATXN1, B4GALT1, BCL2A1, BCL3, CCDC69, CD44, CD55, CD58, CDK14, CDKN2D, CFLAR, CPD, CRISPLD2, CSNK1D, DENND5A, DUSP1, DYNLT1, EPAS1, FCGR2A, FPR1, FUT7, GAB2, GK, GPSM2, HGF, IER3, IFNGR1, IFRD1, IFT20, IP6K1, JAK2, KIAA0930, KIF1B, KIF3C, KLF5, KLF6, KLF7, LAMP2, LITAF, LRP10, MRPL12, NAMPT, NFKB1, NUMB, PDLIM7, PGS1, PHTF1, RAB31, RABGEF1, RAP2C, RBM47, RIN3, SAMSN1, SAT1, SERPINB1, SH3GLB1, SLC19A1, SMPDL3A, SSH1, STAT3, STX3, TESK2, THBS1, TNFRSF12A, TPM4, TRIB1, TUBA4A, UBE2B, UBE2H, WDR1, WIPI1, ZFP36*
Betamethasone	Corticosteroid	3.96E-03	12	*ACSL1, AREG, DUSP1, FKBP5, GLUL, HPGD, IL1R2, IRAK3, IRS2, NFE2L2, SAMSN1, THBS1*
Pimozide	Antipsychotic	1.22E-02	27	*AREG, CD55, CEBPB, DUSP1, ETS2, FOS, GADD45B, H3C6, HIF1A, ICAM1, IER2, JUN, JUNB, KLF6, LDLR, MAPK1, NCF1C, NDRG1, NFIL3, NFKBIA, NTSR1, PNRC1, PRL, RXRA, SAT1, TRIB1, ZFP36*
Flunisolide	Corticosteroid	1.31E-02	13	*ACSL1, AREG, CD163, CSGALNACT1, DUSP1, ENC1, FKBP5, FPR1, IL1R2, PER1, SAMSN1, THBS1, TPST1*
Ribavirin	Antiviral	1.31E-02	12	*ACSL1, AREG, CD163, DUSP1, FKBP5, FPR1, IL1R2, IMPDH1, PER1, SAMSN1, THBS1, TPST1*
Mefloquine	Antimalarial	1.31E-02	32	*BCL3, CD55, CEBPB, CSGALNACT2, CYP1B1, DUSP1, FOS, FPR1, GADD45B, GCLM, GK, H3C6, HGF, ICAM1, IER2, IRS2, JUN, JUNB, KLF6, LDHA, LDLR, MAP1LC3B, NFIL3, NFKBIA, NIBAN1, PER1, PIM1, PNRC1, SAT1, STX3, TRIB1, ZFP36*
Fluorometholone	Corticosteroid	2.65E-02	11	*ACSL1, AREG, CPD, CYP1B1, DUSP1, FKBP5, FPR1, IL1R2, NFKBIA, PER1, SAMSN1*
Dexamethasone	Corticosteroid	3.60E-02	59	*ABCC2, ACSL1, AREG, CD163, CD44, CD53, CFLAR, CPD, CYP1B1, DUSP1, ETS2, FBRS, FGF13, FKBP5, FPR1, GRB2, GSR, HGF, HIF1A, HPD, HPGD, ICAM1, IL18, IL1R2, IL6R, INPP1, LDHA, LYN, MAOA, MAPK1, MAPK3, NAMPT, NCF2, NFE2L2, NFIL3, NFKB1, NFKBIA, PER1, PRCP, PRL, RARA, RIPOR2, RXRA, SDCBP, SERPINA1, SERPINB2, SLA, SLC22A1, SLCO4C1, SRGN, STAT3, STAT5B, TGFA, TGFB1, THBS1, TLE4, TLR2, TUBA4A, VIM*
Oxprenolol	Beta-blocker	3.85E-02	6	*HIRA, MAPK1, MAPK3, PPP3R1, RAD23B, SLC22A1*
Risedronate	Bisphosphonate	3.85E-02	6	*CDC42, FDPS, MAPK1, MAPK3, RAC1, RHOA*
Sulfasalazine	Disease-modifying anti-rheumatic drug	3.85E-02	13	*ABCC2, BAX, HGF, HIF1A, HPGD, ICAM1, LDHA, MTX1, NFE2L2, NFKB1, NFKBIA, TBXAS1, TPM3*
Flecainide	Antiarrhythmic	4.60E-02	7	*ASAH1, FOS, GDPD3, PGK1, SCN5A, SLC22A1, WIPI1*
Rimexolone	Corticosteroid	4.88E-02	15	*AREG, CYP1B1, DUSP1, FAM53C, FGR, FKBP5, FPR1, IL1R2, JUN, NDRG1, PER1, PFKFB3, SLC11A1, THBS1, TPST1*

Upregulated persistent genes unique to non-survivors were used for enrichment. Drug-gene sets from DSigDB were significantly enriched if they had a Benjamini-Hochberg adjusted p-value <0.05 and q-value <0.2, based on the default settings of the enricher function in clusterProfiler. Only FDA-approved drugs were used for enrichment (1202 drugs with bioassay results).

**Figure 5:**
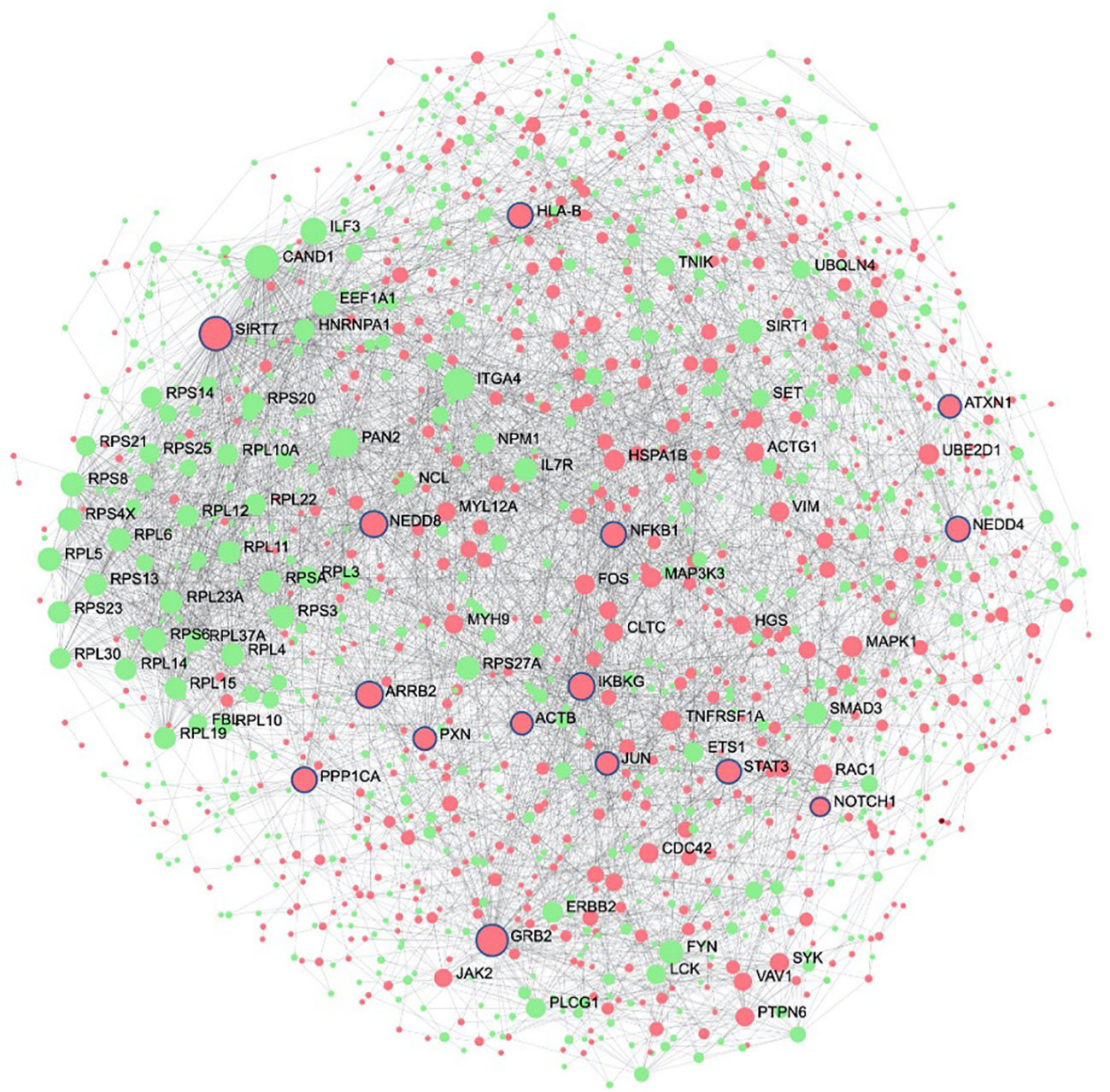
Protein interaction network of persistent genes in deceased patients highlight potential hub genes for drug targeting. Zero-order (i.e., only dysregulated nodes) functional protein-protein interactions (PPI) networks were drawn using NetworkAnalyst. Dots represent nodes (genes and their protein products) and are coloured red for upregulated and green for downregulated. Lines joining the dots represent known PPI from InnateDB. Hub genes, which are genes with multiple interactions, are displayed as the largest nodes (size related to hub degree) and are labelled. Hub genes are attractive targets for drugs as they are expected to regulate or interact with multiple other dysregulated genes and proteins during severe disease. Drugs with known interactions with the top 15 upregulated hubs (circled in blue) are listed in **Table 3**.

The second systems-biology approach was based on network analysis of persistent genes only found in non-survivors (**Figure 5**). Hub genes/proteins, which are genes in a network with multiple connections to other genes/proteins, are attractive druggable targets since they are expected to drive biology by regulating and/or interacting with multiple dysregulated genes/proteins. The top 15 most interconnected upregulated hub genes were *SIRT7*, *GRB2*, *ARRB2*, *IKBKG*, *NEDD8*, *PPP1CA*, *HLA-B*, *NEDD4*, *STAT3*, *NFKB1*, *PXN*, *JUN*, *ATXN1*, *ACTB*, and *NOTCH1* (**Figure 5**). Approved pharmaceutical drugs that target these hub genes were obtained from *genecards.org*, which sources information from a variety of databases including DrugBank, PharmGKB, DGIdb, and Novoseek (**Table 3**). The drug that covered the most hub genes was dexamethasone (6/15 hub genes), followed by the immunomodulator cyclosporine and proteasome inhibitor bortezomib (4/15 hub genes), and a variety of drugs with immunomodulatory function were also identified (*e.g.*, tacrolimus, thalidomide, sulfasalazine, and infliximab). Interestingly, *ICAM1*, which was the only persistent gene seen in both GWAS of sepsis and COVID-19 (**Table S2**), has known protein-protein interactions with the two hub genes *NFKB1* and *STAT3* and was also part of drug-gene sets of aspirin, thioridazine, pimozide, mefloquine, dexamethasone, and sulfasalazine (**Table 2**), suggesting these repurposed drugs may also modulate *ICAM1*. Several of the top persistently downregulated hub genes were ribosomal proteins (*RPS6*, *RPS8*, *RPL5*) indicating that ribosomal dysfunction might be another area to target therapeutically (**Figure 5**). Other persistently downregulated hub genes included *CAND1*, *ITGA4*, *PAN2*, *ILF3*, *EEF1A1*, *SIRT1*, *FYN*, *IL7R*, *SMAD3*, and *NCL*; finding drugs that can activate these hub genes/proteins may also be useful therapeutically.

**Table 3:** Drugs that target hub genes in network of persistent genes in deceased patients.

Gene	Description	Degree	Drugs targeting the Hub proteins
*SIRT7*	sirtuin 7	141	Nicotinamide
*GRB2*	growth factor receptor bound protein 2	109	Pegadamase, Carbamoylcholine, Dexamethasone, Dopamine, Estradiol, Inositol, Letrozole, Tamoxifen
*ARRB2*	arrestin beta 2	70	Buprenorphine, Fentanyl, Methamphetamine, Tramadol, Dopamine, Isoprenaline, Morphine
*IKBKG*	inhibitor of nuclear factor kappa B kinase regulatory subunit gamma	66	Artesunate, Primaquine, Pyrimethamine, Sulfadoxine, Zinc
*NEDD8*	NEDD8 ubiquitin like modifier	65	NA
*PPP1CA*	protein phosphatase 1 catalytic subunit alpha	61	Cantharidin, Cyclosporine, Tacrolimus, Phosphoric acid
*HLA-B*	major histocompatibility complex, class I, B	55	Abacavir, Carbamazepine, Acetazolamide, Amoxicillin, Carbimazole, Clavulanic acid, Clindamycin, Dapsone, Flucoxacillin, Fosphenytoin, Methazolamide, Methimazole, Minocycline, Oxcarbazepine, Pazopanib, Phenytoin, Ribavirin, Sulfasalazine, Ticlopidine, Trichloroethylene, Allopurinol, Benzylpenicillin, Infliximab, Interferon-beta-1a, Lamotrigine, Nevirapine, Peginterferon alfa, Phenobarbital, Phenoxymethylpenicillin, Propylthiouracil, Clozapine, Lamivudine, Stavudine, Thalidomide, Antipyrine, Busulfan, Chenodeoxycholic acid, Chlorzoxazone, Cholesterol, Cimetidine, Cyclosporine, Dexamethasone, Tacrolimus, Testosterone, Tolbutamide, Ursodeoxycholic acid, Iron, Zinc
*NEDD4*	NEDD4 E3 ubiquitin protein ligase	49	Warfarin, Phosphoric acid, Pyrophosphoric acid
*STAT3*	signal transducer and activator of transcription 3	48	Niclosamide, Rituximab, Acitretin, Acalabrutinib, Amphotericin B, Clotrimazole, Durvalumab, Miconazole, Tremelimumab, Omacetaxine mepesuccinate, Celecoxib, Digoxin, Ouabain, Pyrimethamine, Bortezomib, Cholesterol, Cisplatin, Curcumin, Cyclosporine, Dactinomycin, Dasatinib, Dexamethasone, Docetaxel, Doxorubicin, Erlotinib, Filgrastim, Gefitinib, Heparin, Imatinib, Iron, Losartan, Metformin, Mifepristone, Paclitaxel, Parthenolide, Ribavirin, Rosiglitazone, Rosuvastatin, Sirolimus, Sorafenib, Sulindac, Tamoxifen, Testosterone, Thalidomide, Valsartan
*NFKB1*	nuclear factor kappa B subunit 1	48	Sulfasalazine, Donepezil, Glycyrrhizic acid, Racephedrine, Triflusal, Benfotiamine, Deoxycholic acid, Erdosteine, Artesunate, Baclofen, Bortezomib, Chlorambucil, Chlorpropamide, Disulfiram, Hydrocortisone, Hydroquinone, Masoprocol, Mitoxantrone, Nifedipine, Parthenolide, Protriptyline, Rutin, Sulfaphenazole, Thalidomide, Triamcinolone, Etoposide
*PXN*	paxillin	46	Lovastatin, Acetylcholine, Carbamoylcholine, Cholesterol, Colchicine, Dasatinib, Dexamethasone, Doxycycline, Heparin, Losartan, Potassium, Progesterone, Valproic acid
*JUN*	Jun proto-oncogene, AP-1 transcription factor subunit	45	Vinblastine, Adapalene, Irbesartan, Racephedrine, Atomoxetine, Bupropion, Cinnarizine, Ciprofibrate, Clofibrate, Clotrimazole, Colchicine, Cupric chloride, Diphenhydramine, Fenofibrate, Gemfibrozil, Methimazole, Quinapril, Sertraline, Tropisetron, Lipoic acid, Anethole, Bexarotene, Bortezomib, Cerivastatin, Chenodeoxycholic acid, Chloramphenicol, Curcumin, Cyclosporine, Cytarabine, Dactinomycin, Dexamethasone, Dicoumarol, Etoposide, Mifepristone, Pioglitazone, Raloxifene, Rosiglitazone, Selenious acid, Sirolimus, Tamoxifen, Troglitazone
*ATXN1*	ataxin 1	42	Testosterone
*ACTB*	actin beta	41	Cyclophosphamide, Ethinylestradiol
*NOTCH1*	notch receptor 1	40	Dexamethasone, Bortezomib, Everolimus, Hydrocortisone, Mercaptopurine, Methotrexate, Paclitaxel, Prednisolone, Ribociclib, Temozolomide, Doxorubicin, Doxycycline, Sirolimus

The top 15 upregulated hub genes, based on hub degree (how many interactions it had in the network) are listed below. Approved drugs that target these genes were obtained from genecards.org, which sources information from a variety of databases including DrugBank, PharmGKB, DGIdb, and Novoseek, with evidence of interaction or an inferred relationship. The protein interaction network is in **Figure 5**.

## 4 Discussion

By analyzing COVID-19 and non-COVID-19 sepsis patients longitudinally, we showed that, based on gene expression data, persistent immune dysfunction occurred in patients who eventually died, while partial immune resolution occurred in survivors, regardless of COVID-19 status. This persistent immune dysfunction involved both inflammatory and immunosuppressive components (**Figures 1C**, **2**, **3A**), as observed in both COVID-19 and non-COVID-19 sepsis non-survivors (**Figure S2C, D**) and in external datasets of COVID-19 and sepsis patients (**Figure S4**). While differences in the early antiviral response existed between these two groups (**Figures S2B, S9**), consistent with our previous work (11), overall, the underlying persistent immune dysfunction involved in mortality was highly conserved in both COVID-19 and non-COVID-19 sepsis (**Figure S2D**). The connection between persistence and mortality was further supported by persistent enrichment of our published mortality signature (**Figure 4A**) and the results from the endotype analysis, where most non-survivors remained associated with the high severity endotypes NPS and INF throughout disease (**Figure 4C**).

Persistently dysregulated inflammatory processes included inflammatory processes involving IL-1, IL-6, TNFα, and complement that failed to resolve in patients who died (**Figures 1**, **2**). Interestingly, IL-4 and IL-13 signaling was also persistently upregulated in non-survivors (**Figures 1**, **2**); this could be reflective of a transition towards Type-2 immunity, which could occur during increased pathogen burden (51) and is associated with poor outcomes in sepsis (52). In addition, this shift could be reflective of cellular reprogramming (CR): the process by which innate immune cells such as monocytes or macrophages lose their ability to respond appropriately to pathogens (53, 54), which could be highly detrimental during infection. CR macrophages have certain properties aligned with the Type-2 immunity associated M2 macrophage phenotype, and we previously showed that a CR gene signature predicted severe sepsis and organ failure (55). Adaptive immune deficits centered around persistent T-cell dysfunction in non-survivors, but reversal/correction of this dysfunction occurred in survivors (**Figure 3**). Overall, this persistence was consistent with the general concept of PICS, but this syndrome has only been described to date by using blood cytokine/protein markers and changes in specific cell populations (56). This is the first gene expression study showing that persistent gene expression analogous to PICS occurred similarly in both sepsis and severe COVID-19 ICU patients with worse outcomes.

Currently, it is unclear what enables some patients to correct their immune dysfunction, although this could involve individual factors such as natural immunity, predisposing conditions, or underlying patient genetics. Additionally, the idea of persistence is consistent with epigenetic mechanisms, and multiple immune genes were found both in persistent genes and differentially methylated genes in sepsis and COVID-19 (**Figure S3**). However, since non-survivors had persistently dysregulated pathways that resolved in survivors, this indicated that there may be treatable traits and pharmacological methods that could reverse persistence and decrease mortality. Currently-approved and in-trial immunomodulatory therapies for COVID-19 include corticosteroids such as dexamethasone (21), IL-6 signaling inhibitors such as tocilizumab (57) and baricitinib (23), the recombinant IL-1 receptor antagonist anakinra (58), and the complement inhibitor vilobelimab (59); these all target enriched pathways/gene sets that were observed to be persistently dysregulated in non-survivors (“Inflammatory response”, “IL-6 JAK STAT3 signaling”, “IL-1 signaling”, and “Complement”) (**Figures 1**, **2**). Thus, targeting persistent genes and mechanisms appears to be a valid approach to find additional therapies. The identification of dexamethasone, a known effective treatment for COVID-19 (and for subsets of septic shock (60)), using both drug-gene set enrichment (**Table 2**) and network hub genes (**Table 3**), supported the validity of these methods for identifying potential repurposed drugs for sepsis and COVID-19. Other potential immunomodulators that could address the persistent inflammation included aspirin, sulfasalazine, and cyclosporine. There were also other drugs identified without evident immunomodulator functions, including the anti-psychotic thioridazine and the anti-arrhythmic flecainide, which have demonstrated survival benefits in mouse and rat sepsis models, respectively, likely through off-target inhibition of the NF-κB pathway (61, 62). These identified drugs should be further assessed *in vitro*, *in vivo*, and in clinical trials for potential efficacy.

While these identified drugs primarily dampen inflammatory mechanisms, it is also important to address the adaptive immune deficits observed in this cohort, since only focusing on anti-inflammatory therapies has not proven to be successful in sepsis (13). Thus, treatments that aim to restore adaptive suppression, focusing on T-cell functions/numbers should also be considered. They could be potentially concurrently administered with anti-inflammatories, or selectively applied to patients that require more immune stimulation rather than anti-inflammation based on underlying gene expression profiles or clinical variables. Therapies that addresses adaptive immune suppression could include checkpoint inhibitors (63) and IL-7 (64), which are currently being evaluated for sepsis and perhaps should also be considered for severe COVID-19. For example, *IL7R* was one of the top downregulated hub genes (**Figure 5**), further supporting potential use of IL-7. Monitoring immune function longitudinally throughout hospitalization (*e.g.*, using gene-expression panels, cytokine measurements) could identify patients that fail to resolve immune dysfunction within the first week of ICU hospitalization, which could inform healthcare providers to consider additional care or enrollment into immunomodulatory clinical trials. As shown in this cohort, the time at which a patient is on their disease timeline (in this case, D1 or D7 in the ICU) needs to be considered when evaluating therapies.

There are some limitations to this study. First, these results are from a single cohort of mostly male patients and, while the major finding of persistence has been confirmed by re-analysis of other studies (30, 42) (**Figure S4**), they should be validated in larger, sex-balanced studies. Despite the modest sample size, thousands of DE genes were still identified, suggesting that the study was adequately powered for finding gene expression differences. Critically, these samples were paired with two timepoints, enabling patient indexing, which can help to eliminate various sources of patient heterogeneity and potential confounders that could potentially overshadow true DE changes. It is also possible that bulk RNA-Seq results could be altered by different cell proportions. However, even after adjusting with cell proportion data, similar patterns of differential expression were observed (**Figure S8**). Multiple other potential confounders were also addressed, including corticosteroid use, COVID-19 status (**Figures S2, S9**), and sample imbalance (**Figure S10**), all of which showed no substantial effects on the gene expression patterns observed.

In summary, mortality in both sepsis and COVID-19 was highly associated with persistent immune dysfunction during the first week in the ICU, with both inflammatory and immunosuppressive components. To improve outcomes, these patients require novel immunomodulatory treatments to treat immune dysfunction throughout the ICU stay, with multiple immunomodulatory drug candidates identified for further *in vitro* and *in vivo* testing.

## Data availability statement

The datasets presented in this study can be found on the Gene Expression Omnibus (https://www.ncbi.nlm.nih.gov/geo/). The accession numbers are: GSE185263 and GSE222393.

## Ethics statement

The studies involving humans were approved by Research Ethics Boards of St. Michael’s Hospital (“COLOBILI”, REB#20-078) and the University of British Columbia (“COVID-19 Sepsis Study”, REB#H20-02441). The studies were conducted in accordance with the local legislation and institutional requirements. The participants provided their written informed consent to participate in this study.

## Author contributions

AA: Formal Analysis, Investigation, Validation, Visualization, Writing – original draft, Writing – review & editing. AB: Formal Analysis, Validation, Writing – review & editing. PZ: Formal Analysis, Writing – review & editing. RF: Methodology, Writing – review & editing. AL: Formal Analysis, Funding acquisition, Validation, Writing – review & editing. UT: Conceptualization, Writing – review & editing. AJB: Conceptualization, Funding acquisition, Resources, Writing – review & editing. CS: Conceptualization, Funding acquisition, Resources, Writing – review & editing, Project administration, Supervision. RH: Conceptualization, Formal Analysis, Funding acquisition, Investigation, Project administration, Resources, Supervision, Writing – original draft, Writing – review & editing.
