# Supplementary material for: Persistence is key: unresolved immune dysfunction is lethal in both COVID-19 and non-COVID-19 sepsis
Source: Front Immunol. 2023 Sep 26;14:1254873. doi: 10.3389/fimmu.2023.1254873 (PMC10562687; doi:10.3389/fimmu.2023.1254873)
Supplement: Supplementary file 1 [file DataSheet_1.docx]

Supplementary Material

**Persistence is Key: Unresolved Immune Dysfunction is Lethal in Both COVID-19 and non-COVID-19 Sepsis**

Andy Y An, Arjun Baghela, Peter Zhang, Reza Falsafi, Amy H Lee, Uriel Trahtemberg, Andrew J Baker, Claudia C dos Santos, Robert EW Hancock*

*** Correspondence:** Dr. Robert EW Hancock: bob@hancocklab.com

# Supplementary Tables

Table S1. The top 20 up- and down-regulated persistent genes seen in non-survivors.

Log_2_ fold change (FC) is shown of deceased patients vs. healthy controls at D1 and D7. Persistent genes only found in non-survivors are bolded.

| **Upregulated** | | | **Downregulated** | | |
| --- | --- | --- | --- | --- | --- |
| **Gene** | **D1 Log_2_ FC** | **D7 Log_2_ FC** | **Gene** | **D1 Log_2_ FC** | **D7 Log_2_ FC** |
| *CYP19A1* | 11.77 | 8.73 | *UTS2* | -7.86 | -7.73 |
| ***ADAMTS3*** | 10.16 | 7.89 | ***SLC4A10*** | -5.71 | -4.31 |
| ***NECAB1*** | 9.52 | 8.23 | ***FCER1A*** | -4.93 | -4.39 |
| *ADAMTS2* | 8.60 | 8.60 | ***MS4A2*** | -5.15 | -3.76 |
| ***CD177P1*** | 9.04 | 7.81 | ***ENPP3*** | -4.52 | -4.35 |
| *METTL7B* | 9.38 | 6.93 | ***HDC*** | -5.13 | -3.34 |
| *OLAH* | 8.65 | 7.60 | ***CACNG6*** | -5.51 | -2.89 |
| ***ENSG00000243144*** | 8.78 | 7.45 | ***UICLM*** | -3.31 | -5.01 |
| ***PCSK9*** | 9.49 | 6.47 | ***DGKK*** | -3.75 | -4.42 |
| *ENSG00000254420* | 8.80 | 7.16 | ***TRAV1-2*** | -4.50 | -3.46 |
| *SLC51A* | 8.98 | 6.66 | ***LINC02458*** | -3.87 | -4.05 |
| *CD177* | 8.24 | 7.15 | ***CPA3*** | -4.66 | -3.21 |
| *PCOLCE2* | 9.12 | 6.24 | ***MEG3*** | -3.60 | -4.23 |
| *MMP8* | 9.03 | 6.21 | ***EIF4BP6*** | -3.94 | -3.75 |
| *ZDHHC19* | 8.68 | 6.54 | ***LRRN3*** | -4.10 | -3.27 |
| *OLFM4* | 7.25 | 7.81 | ***AKAP12*** | -4.04 | -3.07 |
| *NR2E1* | 7.31 | 7.23 | *NOG* | -3.86 | -3.20 |
| *TREML4* | 7.08 | 7.37 | ***FCGBP*** | -3.76 | -3.18 |
| *GLDN* | 7.22 | 6.81 | ***TRGV3*** | -3.41 | -3.35 |
| *GPR42* | 7.95 | 5.85 | ***DSC1*** | -3.63 | -3.09 |

Table S2. Overlap of persistently dysregulated genes with genes associated with sepsis and COVID-19 severity identified through genome wide association studies (GWAS). Four GWAS studies were performed, two for sepsis (Rosier *et al.* 2021^26^,

Engoren *et al.* 2022^27^) and two for COVID-19 (Kousathanas *et al.* 2022^28^, Pairo-Castineira et al. 2023^29^). Genes that were identified in these studies and were also persistently up- or down-regulated in non-survivors and/or survivors are shown below.

| **Gene** | **Persistent in** | **Directionality** | **Disease** |
| --- | --- | --- | --- |
| *AK5* | Both | Down | COVID-19^29^ |
| *BPI* | Both | Up | Sepsis^27^ |
| *CD274* | Both | Up | Sepsis^27^ |
| *F5* | Both | Up | Sepsis^27^ |
| *ICAM5* | Both | Up | COVID-19^28^ |
| *IGF1* | Both | Up | Sepsis^27^ |
| *LTF* | Both | Up | Sepsis^27^ |
| *LZTFL1* | Both | Down | COVID-19^28,29^ |
| *MUC1* | Both | Up | COVID-19^28^ |
| *PLSCR1* | Both | Up | COVID-19^28,29^ |
| *PRTN3* | Both | Up | Sepsis^27^ |
| *SDC1* | Both | Up | Sepsis^27^ |
| *SLC28A3* | Both | Up | Sepsis^26^ |
| *TIMP2* | Both | Up | Sepsis^27^ |
| *TLR5* | Both | Up | Sepsis^27^ |
| *ADGRE2* | Non-survivors | Up | Sepsis^27^ |
| *ARHGAP27* | Non-survivors | Up | COVID-19^28^ |
| *ATP11A* | Non-survivors | Up | COVID-19^28,29^ |
| *BCKDK* | Non-survivors | Up | Sepsis^27^ |
| *C1GALT1C1* | Non-survivors | Up | COVID-19^28^ |
| *C3orf18* | Non-survivors | Down | Sepsis^26^ |
| *CACNA2D2* | Non-survivors | Down | Sepsis^26^ |
| *DPH7* | Non-survivors | Down | Sepsis^26^ |
| *DPP4* | Non-survivors | Down | Sepsis^27^ |
| *DPYD* | Non-survivors | Up | Sepsis^26^ |
| *F8* | Non-survivors | Up | COVID-19^28^ |
| *GZMK* | Non-survivors | Down | Sepsis^27^ |
| *HEMK1* | Non-survivors | Down | Sepsis^26^ |
| *HLA-DRA* | Non-survivors | Down | Sepsis^27^ |
| *HPSE* | Non-survivors | Up | Sepsis^27^ |
| *ICAM1* | Non-survivors | Up | Sepsis^27^, COVID-19^28^ |
| *IL10RB* | Non-survivors | Up | COVID-19^28,29^ |
| *LINC00649* | Non-survivors | Down | COVID-19^28^ |
| *MLKL* | Non-survivors | Up | Sepsis^27^ |
| *NAMPT* | Non-survivors | Up | Sepsis^27^ |
| *NFKB1* | Non-survivors | Up | Sepsis^27^ |
| *NR1H2* | Non-survivors | Up | COVID-19^29^ |
| *PCSK9* | Non-survivors | Up | Sepsis^27^ |
| *PRL* | Non-survivors | Up | Sepsis^27^ |
| *PYGM* | Non-survivors | Up | Sepsis^27^ |
| *RNF135* | Non-survivors | Up | Sepsis^26^ |
| *RPL6* | Non-survivors | Down | Sepsis^26^ |
| *SLFN12L* | Non-survivors | Down | Sepsis^26^ |
| *TBC1D32* | Non-survivors | Down | Sepsis^26^ |
| *THBS3* | Non-survivors | Up | COVID-19^28,29^ |
| *TYK2* | Non-survivors | Up | COVID-19^28,29^ |
| *ADAP2* | Survivors | Up | Sepsis^26^ |
| *MMP1* | Survivors | Up | Sepsis^27^ |
| *SLC2A5* | Survivors | Up | COVID-19^29^ |
| *TGM2* | Survivors | Up | Sepsis^27^ |

# Supplemental Figures


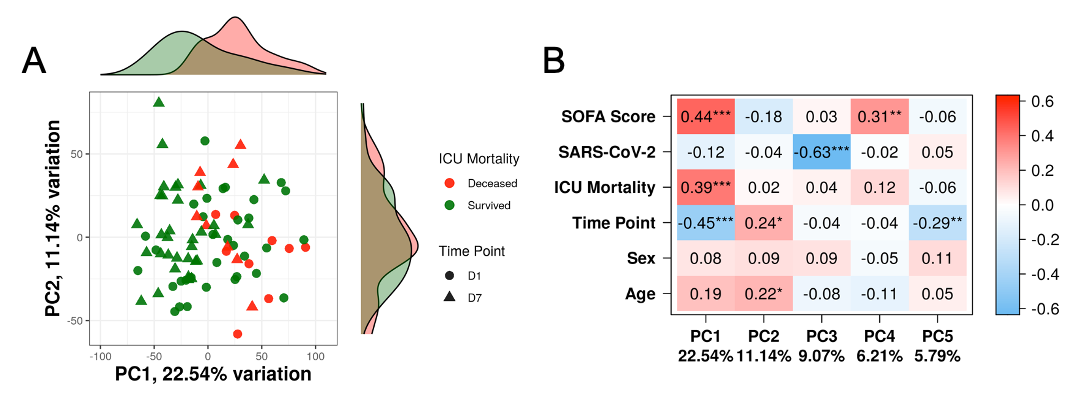


**Figure S1. Eventual ICU mortality was a major contributor to gene expression variation.** **A:** Principal component analysis plot of all ICU samples, with density plots coloured by ICU mortality on the sides. There was visible separation along PC1 (top density plot) for ICU mortality. **B:** Correlation plot of various metadata variables to principal components, with the proportion of variation attributed to each component displayed below. ICU mortality, SOFA score, and timepoint were significantly correlated to the largest principal component, PC1, while SARS-CoV-2 positivity was significantly correlated to the smaller PC3.


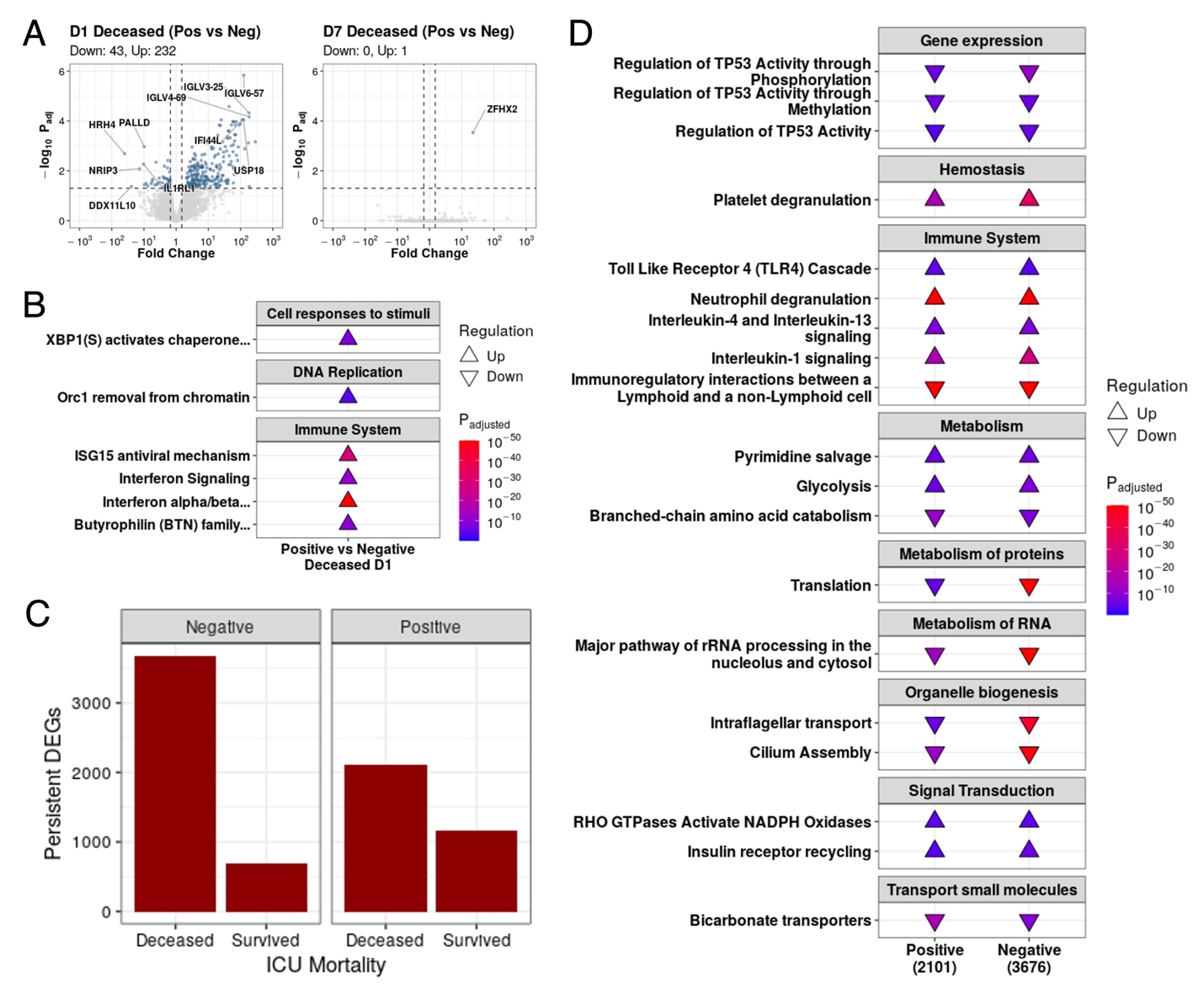


**Figure S2. Analysis of persistent genes when splitting by SARS-CoV-2 positivity. A:** Volcano plots of differentially expressed genes (DEGs) between SARS-CoV-2 positive (Pos) and negative (Neg) patients who eventually died, with the comparison at D1 or D7. **B:** Pathway enrichment of DEGs from the D1 comparison of SARS-CoV-2 positive and negative patients who eventually died. **C:** Numbers of persistent DEGs in patients who eventually died compared to those in survivors, separated by SARS-CoV-2 positivity. In both groups, non-survivors had substantially more persistent genes than survivors. **D:** Shared pathways enriched by persistent genes in non-survivors of SARS-CoV-2 positive and negative patients. The number of persistent genes in each group is displayed under each label.


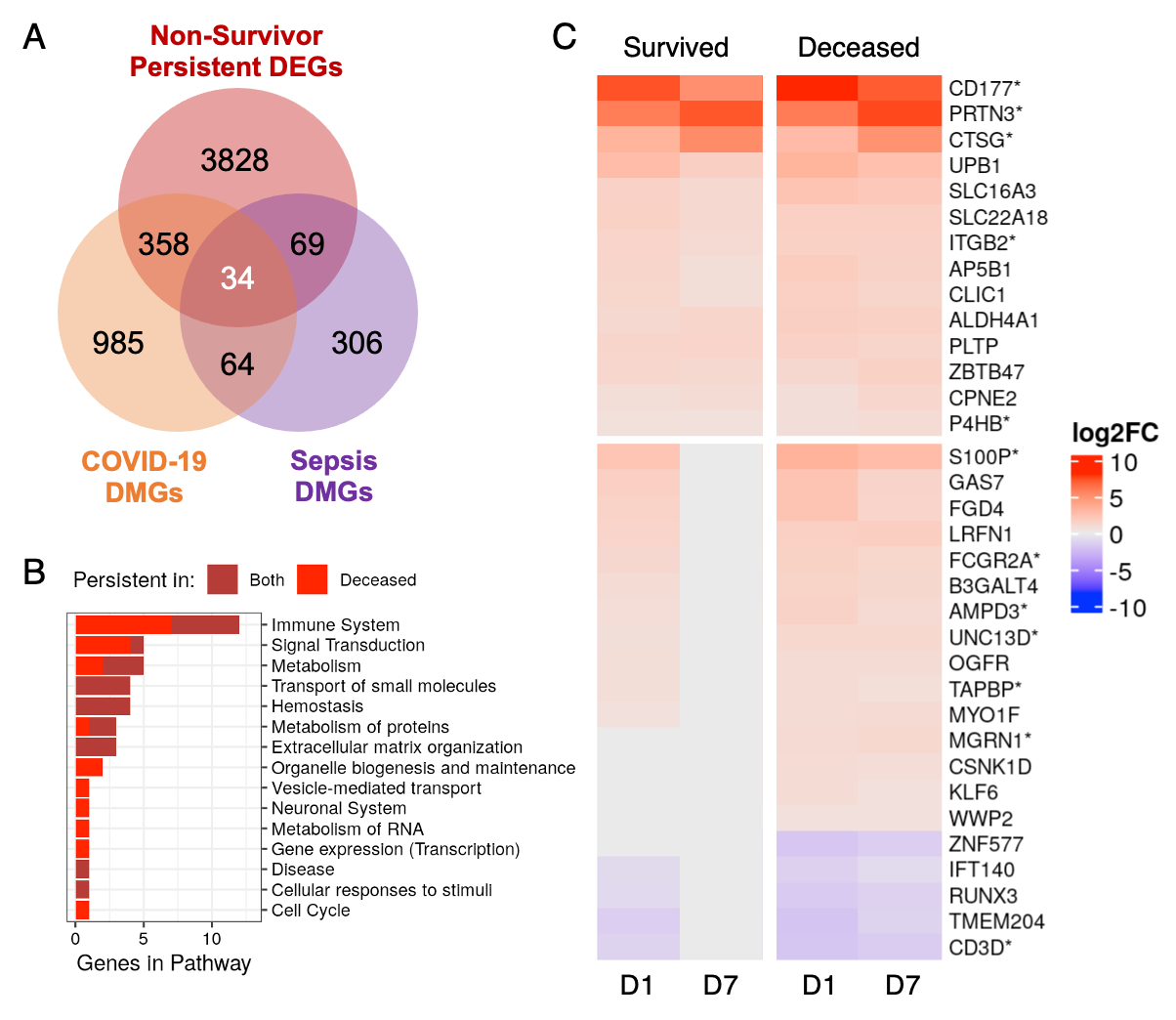


**Figure S3. Persistent genes in non-survivors overlapped with DNA methylation datasets of sepsis and COVID-19.**

**A:** Overlaps between the 4,289 persistent differentially expressed genes (DEGs) in eventually deceased patients, 473 differentially methylated genes (DMGs) from a sepsis DNA methylation dataset from Binnie et al. 2020^34^, and 1,441 DMGs from two COVID-19 DNA methylation datasets: Balnis et al. 2021^36^ and Castro de Moura et al. 2021^35^. Thirty-four genes were found in all three groups. **B:** Reactome pathway groups that each of the 34 overlapped genes belonged in (some genes belonged in more than one pathway group). Genes were coloured based on if they were persistent in non-survivors or all patients. Notably, the pathway associated with the most genes was “Immune System”. **C:** Fold change heatmap of the 34 overlapping genes at D1 and D7 in non-survivors and/or survivors. Shading in the heatmap represents log_2_ fold change (log2FC). Only DE genes are shown. The asterisk beside the gene indicates if it was part of the Immune System Reactome pathway group.


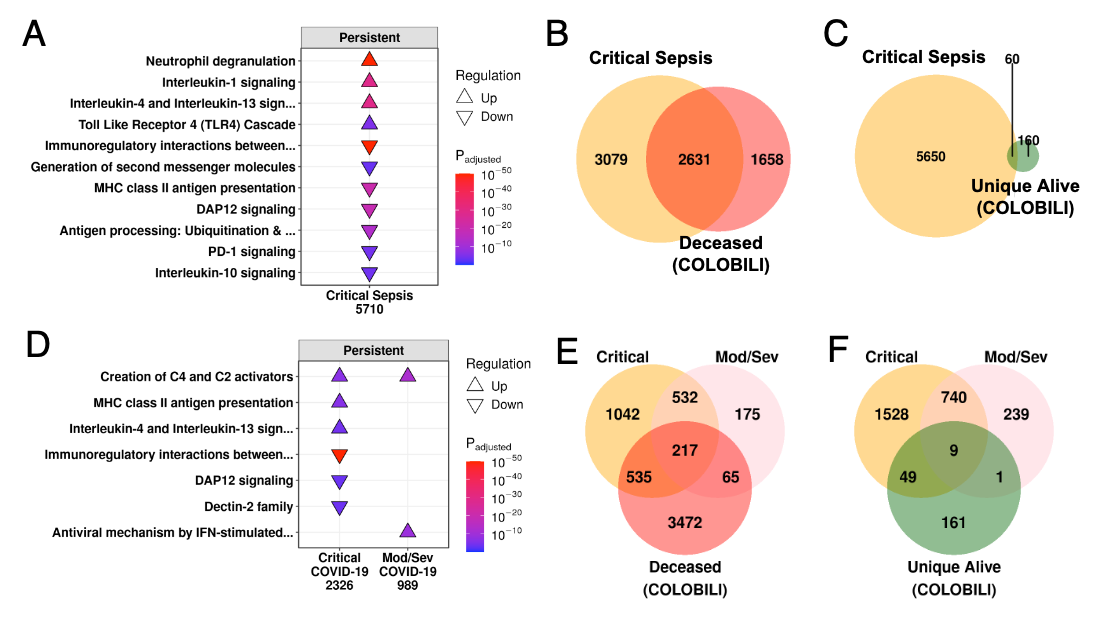


**Figure S4. Substantial numbers of persistent genes were found in higher severity patients in two validation cohorts of sepsis and COVID-19.** **A:** GSE196117 was a collection of sepsis patients that were enrolled as part of a clinical study on the efficacy of clarithromycin for sepsis, with peripheral blood collected at day 1 (18 patients) and day 5 (15 patients) of hospital admission in critically ill sepsis patients, with 7 healthy controls for baseline. Enriched “Immune System” Reactome pathways using the 5,710 persistent genes in critically ill sepsis patients are shown, with the number of persistent genes under each label. A large overlap (**B**) of persistent genes of critically ill sepsis patients in GSE196117 and persistent genes from non-survivors in the COLOBILI cohort was observed, while a substantially smaller overlap (**C**) of persistent genes in GSE196117 and unique persistent genes from survivors in the COLOBILI cohort (*i.e.,* persistent genes in survivors that were not found in deceased patients) was observed. **D:** GSE161918 was a collection of COVID-19 patients with peripheral blood collected within the first 3 days of hospital admission (22 patients) and within 6-11 days after hospital admission (18 patients), with 11 healthy controls. Severity was defined based on the NIH Clinical Spectrum of SARS-CoV-2 Infection, reflective of the level of respiratory support needed. Enriched “Immune System” Reactome pathways using persistent genes in Critical and Moderate/Severe (Mod/Sev) COVID-19 patients, with the number of persistent genes under each label. Not only were there substantially greater numbers of persistent genes in the higher severity group, the persistent genes in Critical patients also substantially overlapped (**E**) with those from the COLOBILI cohort, while a smaller overlap (**F**) was seen with persistent genes from Moderate and Severe COVID-19 patients.


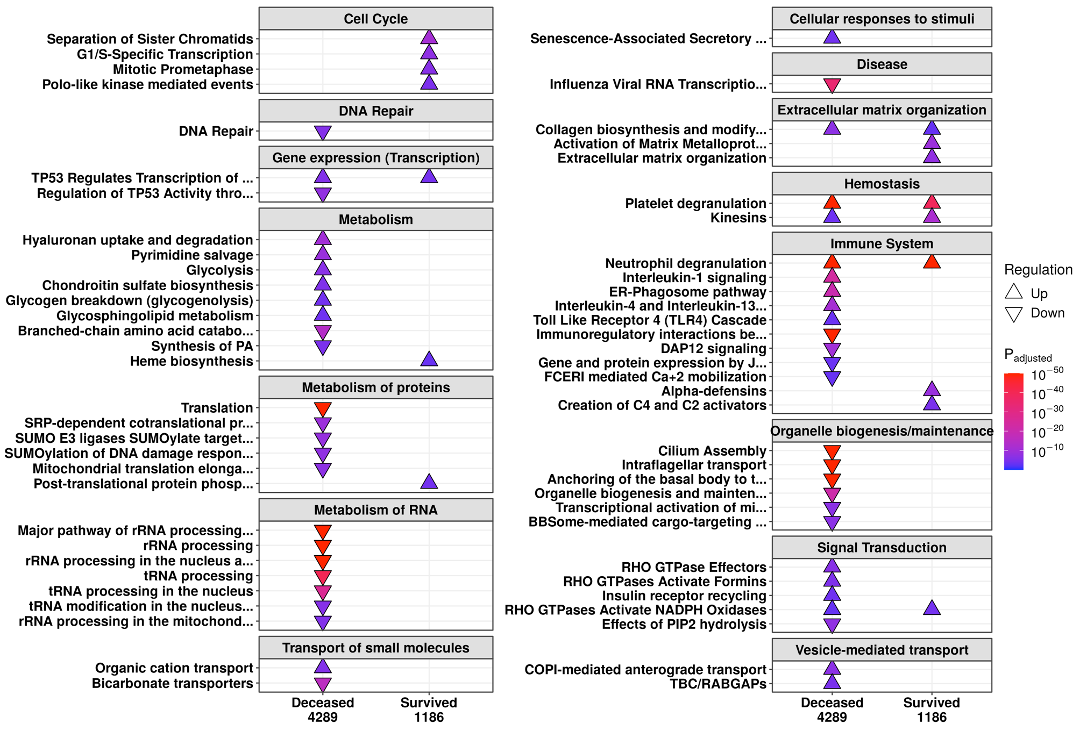


**Figure S5. Eventually deceased patients had persistent dysfunction in multiple cellular pathways.** All enriched Reactome pathways from persistently upregulated (Δ) and downregulated (∇) genes in patients who eventually were deceased or survived are shown. The total numbers of persistent genes in each comparison are under each label.


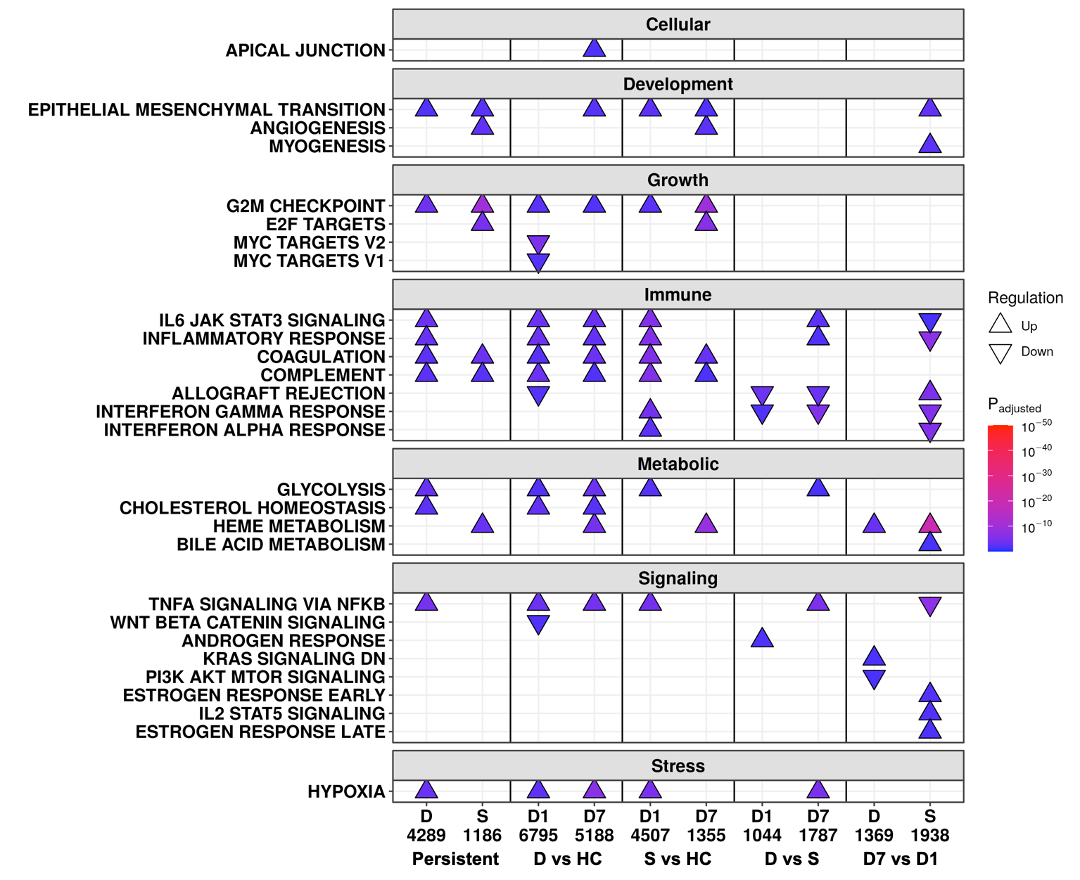


**Figure S6. Enrichment of Hallmark gene sets using persistent and DE genes from multiple comparisons.** All enriched Hallmark gene sets from upregulated (Δ) and downregulated (∇) persistent or DE genes are shown from multiple comparisons: Deceased (D) vs Healthy Controls (HC), Survivors (S) vs HC, D vs S, and Day 7 (D7) vs Day 1 (D1). The total numbers of persistent or DE genes in each comparison are under each label.


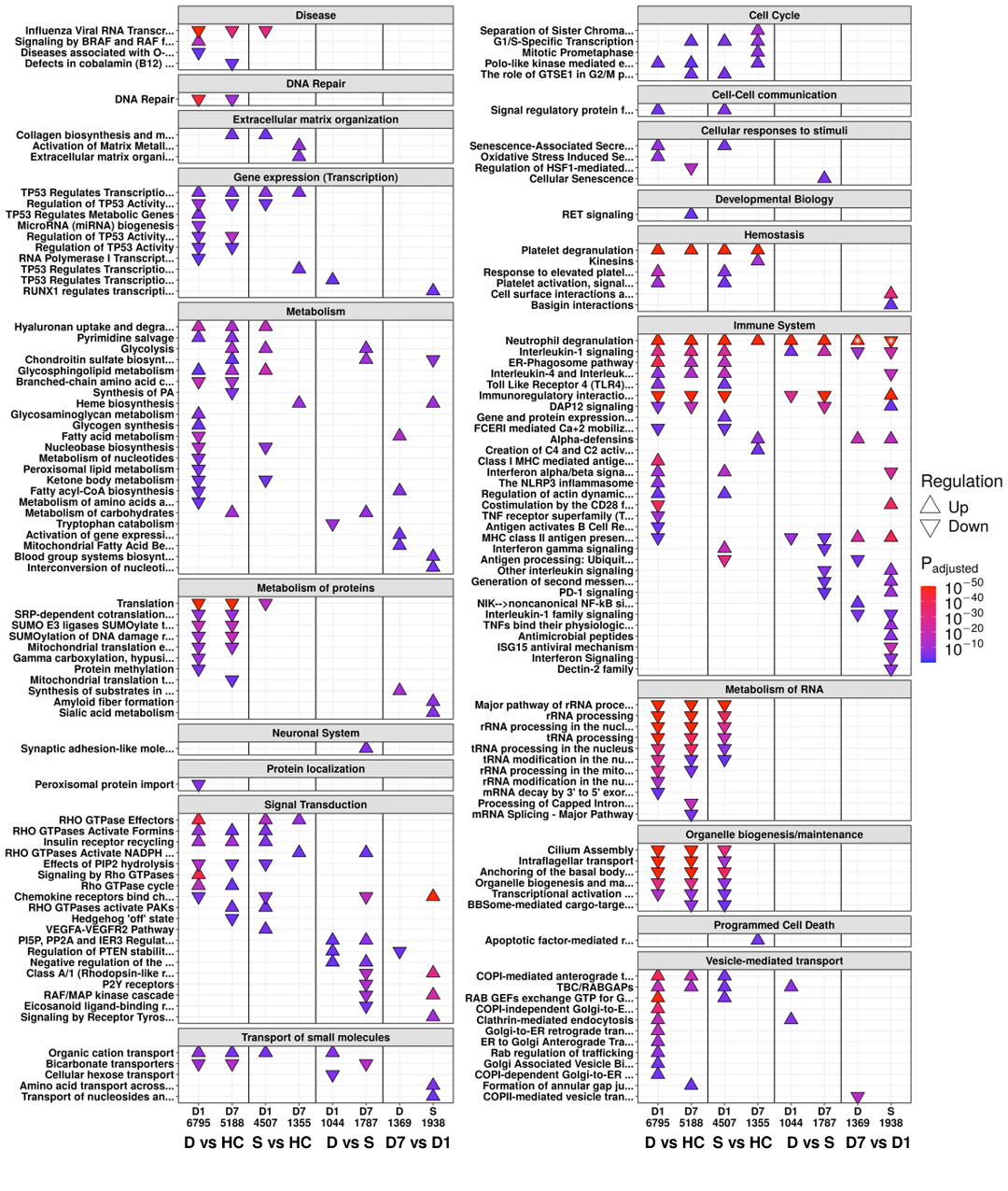


**Figure S7. Enrichment of Reactome pathways using DE genes from multiple comparisons.** All enriched Reactome pathways from upregulated (Δ) and downregulated (∇)DE genes are shown from multiple comparisons: Deceased (D) vs Healthy Controls (HC), Survivors (S) vs HC, D vs S, and Day 7 (D7) vs Day 1 (D1). The total numbers of persistent or DE genes in each comparison are under each label.


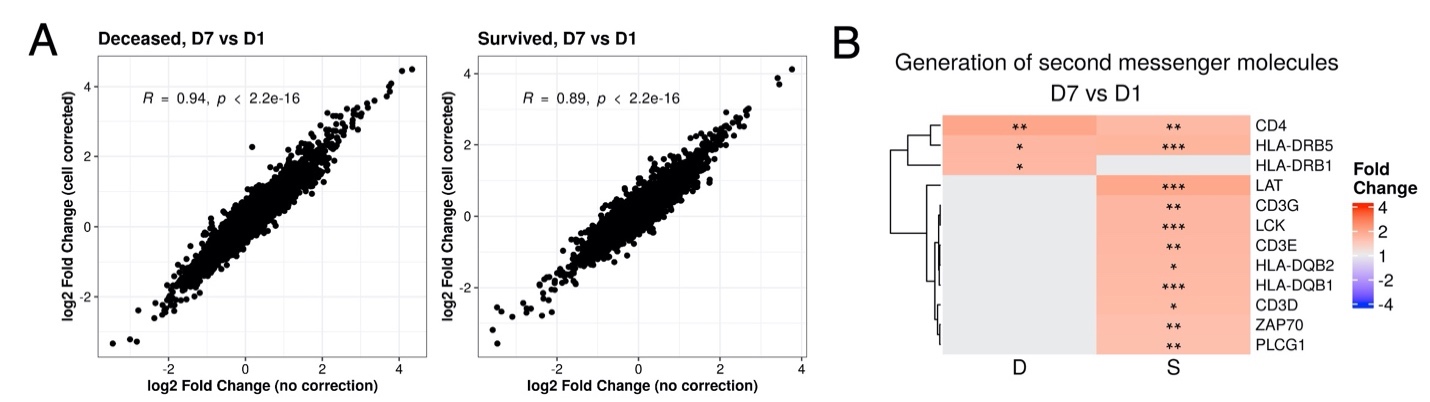


**Figure S8. Cell proportion was not a major contributor to gene expression variation.** **A:** Pearson correlation of the log2 fold change before and after cell proportion correction for each gene in deceased and survived patients over time. High correlation suggested that gene expression variation cannot be well explained by cell proportion differences. Cellular proportions were estimated by CIBERSORTx, a cell deconvolution technique using gene expression data^44^. PCA was performed on CIBERSORTx estimated cell proportion output, and the first two principal components (accounting for 84% of the variance in cell proportions) were added to the DESeq2 model matrix to account for differences in gene expression potentially due to cellular compositional differences. Principal components instead of individual cell type proportions were used to reduce co-linearity issues as well as over-fitting of the model from too many covariates added to the model matrix. **B:** Fold change of genes of interest over time in deceased (D) and surviving (S) patients after cell proportion correction. Even after adjusting for cellular proportions, genes involved in T cell signaling were still upregulated over time only in survivors. *** = p<0.001, ** = p<0.01, and * = p<0.05.


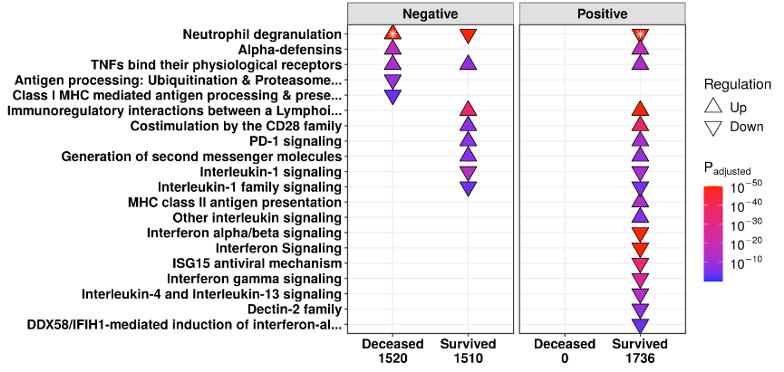


**Figure S9. Only survivors, regardless of SARS-CoV-2 positivity, demonstrated adaptive immune restoration and inflammation resolution over time.** Pathway enrichment was performed on DE genes between D7 and D1 in each patient group, with the number of DE genes in each comparison below each label. Enriched Reactome “Immune System” pathways are shown.


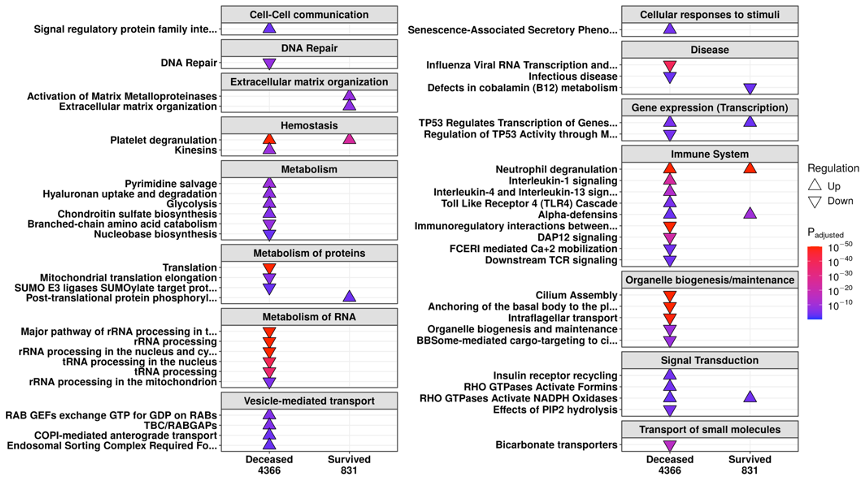
 **Figure S10. Matching patients still resulted in deceased patients having substantially more persistent genes and dysfunction.** The 9 non-survivors were matched to 9 survivors by age (±10 years), sex, COVID-19 status, and sequencing batch. Shown are enriched Reactome pathways from persistent genes, with the number of persistent genes under each label.
